# Minimally invasive retrograde esophageal endoscopic submucosal dissection via gastrostomy using a thin therapeutic endoscope

**DOI:** 10.1055/a-2721-9215

**Published:** 2025-11-19

**Authors:** Tetsuhiko Hirai, Yoichi Yamamoto, Masao Yoshida, Noboru Kawata, Hiroyuki Ono

**Affiliations:** 1 38471 Division of Endoscopy, Shizuoka Cancer Center, Shizuoka, Japan

A 58-year-old woman with dysphagia was diagnosed with advanced esophageal cancer. She achieved complete response with definitive chemoradiotherapy, but cervical esophageal stenosis occurred ( Fig. 1 ). A percutaneous endoscopic gastrostomy was performed for nutritional support. Endoscopic balloon dilation was performed; however, the stricture was refractory, and a standard-caliber endoscope could not pass even after dilation. After dilation, a transnasal endoscope passed through the stricture, and a superficial esophageal carcinoma (Lt/Rw, 0-IIc + IIa, cT1a-MM, 14 mm) was identified distal to the stricture ( Fig. 2 ).

**Fig. 1:**
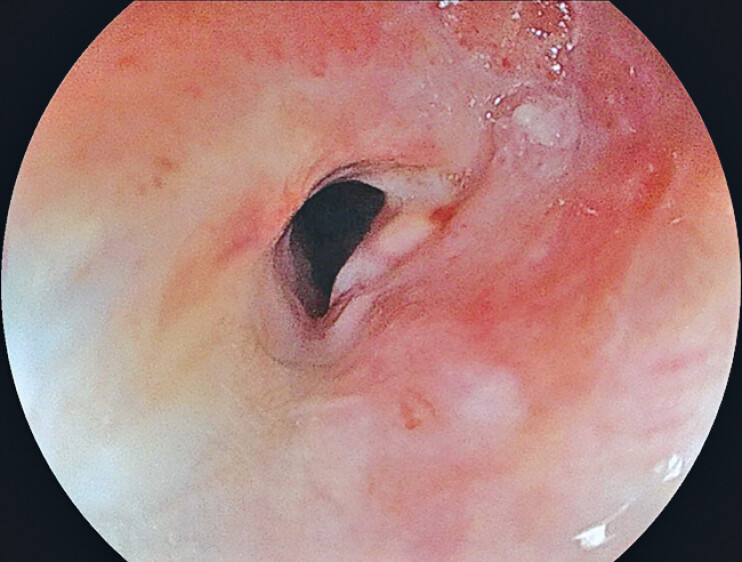
Cervical esophageal stenosis occurred after definitive chemoradiotherapy for advanced esophageal cancer. A standard-caliber transoral endoscope could not pass through.

**Fig. 2:**
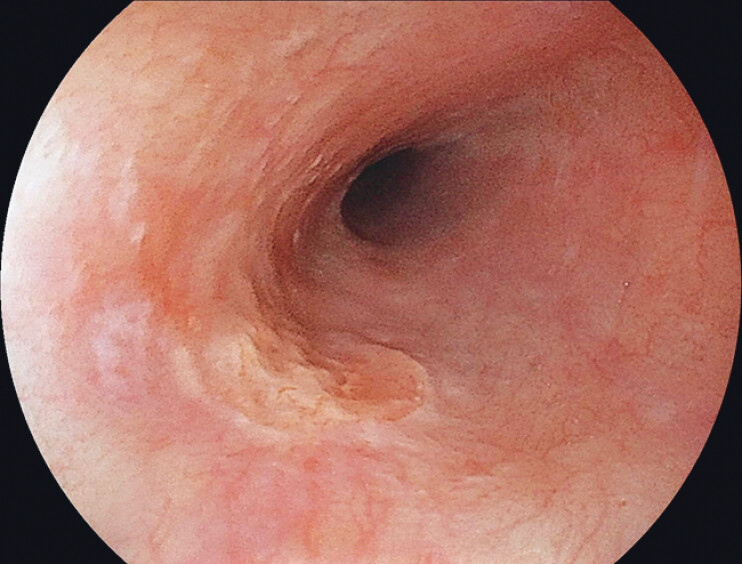
A superficial esophageal carcinoma (Lt/Rw, 0-IIc + IIa, cT1a-MM, 14 mm) identified during dilation for cervical esophageal stenosis.

As passage through the stricture with a standard-caliber endoscope was difficult, retrograde endoscopic submucosal dissection (ESD) via gastrostomy was planned. A thin therapeutic endoscope (EG-840TP; FUJIFILM) was inserted through the gastrostomy without dilation, and successfully provided retrograde access to the esophageal lumen ( Fig. 3 ). Using an ITknife nano (OLYMPUS), ESD was performed without intraoperative complications, and en bloc resection was achieved ( Fig. 4 , Video 1 ). The pathological diagnoses were as follows: squamous cell carcinoma, pT1a-MM, ly0, v0, pHM0, pVM0, and 11 × 10 mm. No local recurrence was observed at 18 months after ESD.

**Fig. 3:**
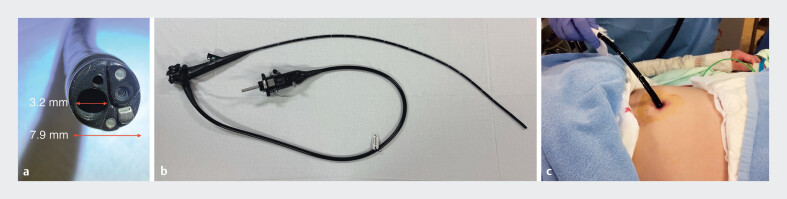
**a** and **b** The EG-840TP (FUJIFILM) is a thin endoscope with an outer diameter of 7.9 mm and a working channel of 3.2 mm, allowing the use of standard therapeutic devices. **c** A thin therapeutic endoscope was inserted through the gastrostomy without dilation for retrograde esophageal access.

**Fig. 4:**
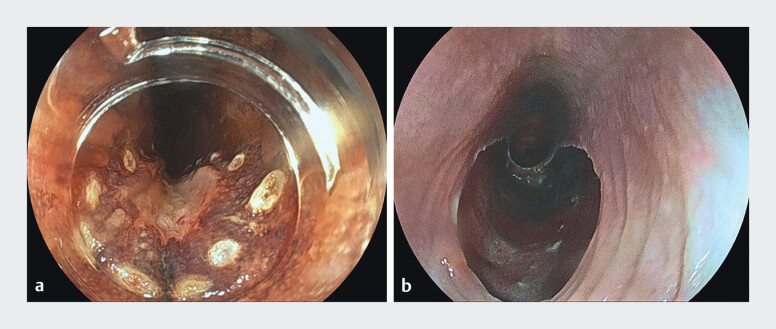
**a** Circumferential marking was performed. **b** Post-ESD ulcer after en bloc resection via a retrograde approach using a thin therapeutic endoscope, with no intraoperative complications.

Retrograde ESD via gastrostomy using a thin therapeutic endoscope achieved en bloc resection of a superficial esophageal carcinoma distal to the stricture, without gastrostomy dilation.Video 1

Retrograde esophageal ESD via gastrostomy, using a standard-caliber therapeutic scope, is a valuable option for patients with difficult transoral insertion, although gastrostomy dilation was required in previous reports 1 2 . Recent studies have also demonstrated the usefulness of thin therapeutic endoscopes in anatomically challenging situations, such as post-treatment esophageal strictures, where conventional scopes are difficult to use 3 4 5 . In this case, because the cervical esophageal stricture existed, the lesion was successfully resected via a retrograde approach using a thin therapeutic endoscope, and without requiring gastrostomy dilation.

A thin therapeutic endoscope enables the use of standard ESD devices and eliminates the need for gastrostomy dilation, thereby simplifying the procedure. This minimally invasive approach may represent a viable therapeutic option for superficial esophageal cancer with severe esophageal strictures.

Endoscopy_UCTN_Code_TTT_1AO_2AG_3AD

## Notes

Endoscopy E-Videos https://eref.thieme.de/e-videos
*E-Videos* is an open access online section of the journal *Endoscopy* , reporting on interesting cases and new techniques in gastroenterological endoscopy. All papers include a high-quality video and are published with a Creative Commons CC-BY license. Endoscopy E-Videos qualify for HINARI discounts and waivers and eligibility is automatically checked during the submission process. We grant 100% waivers to articles whose corresponding authors are based in Group A countries and 50% waivers to those who are based in Group B countries as classified by Research4Life (see: https://www.research4life.org/access/eligibility/ ). This section has its own submission website at https://mc.manuscriptcentral.com/e-videos .
